# Clinical Outcomes of rhBMP-2-Loaded Collagen Sponge for Alveolar Ridge Preservation: A Pilot Study

**DOI:** 10.1055/s-0045-1810610

**Published:** 2025-08-13

**Authors:** Peungchaleoy Thammanichanon, Warisara Ouyyamwongs, Katanchalee Mai-ngam, Pawornwan Rittipakorn

**Affiliations:** 1 Institute of Dentistry, Suranaree University of Technology, Nakhon Ratchasima, Thailand; 2 Oral Health Center, Suranaree University of Technology Hospital, Nakhon Ratchasima, Thailand; 3 Private Practice, Bangkok, Thailand; 4 Metal and Materials Technology (MTEC), Medical Product Innovation Research Team, Prathumtani, Thailand

**Keywords:** alveolar ridge preservation, biomaterials, rhBMP-2, collagen

## Abstract

**Objectives:**

Alveolar bone resorption following tooth extraction poses a significant clinical challenge. While various ridge preservation techniques exist, limitations remain. Recombinant human bone morphogenetic protein-2 (rhBMP-2) delivered via an absorbable collagen sponge (ACS) has shown promise in bone augmentation. This pilot randomized controlled trial aimed to evaluate the efficacy of ACS combined with rhBMP-2 for alveolar ridge preservation in maintaining ridge dimensions after tooth extraction.

**Materials and Methods:**

This pilot randomized controlled trial enrolled 11 participants with 16 extraction sites, randomized to either the intervention (ACS/rhBMP-2) or the control (empty socket) group. The *in vitro* release kinetics of rhBMP-2 from the ACS carrier were measured over 13 days using ELISA (enzyme-linked immunosorbent assay). Clinical procedures involved atraumatic extraction, followed by the placement of ACS/rhBMP-2 in the intervention group. Alveolar ridge dimensions were assessed using cone-beam computed tomography at baseline (T0), and at 4 (T4), 8 (T8), and 12 weeks (T12) post-extraction.

**Statistical Analysis:**

Data were presented as mean ± standard deviation. A linear mixed model was used to compare dimensional changes over time between the two groups. A *p-* value of <0.05 was considered statistically significant.

**Results:**

The *in vitro* assay showed a gradual, sustained release of rhBMP-2 from the ACS, with a cumulative release of 7.28% (117 ± 5.18 µg) over 13 days. No postoperative complications were observed in any participant. At 12 weeks, the ACS/rhBMP-2 group demonstrated a statistically significant preservation of buccal bone height, with 1.80 mm less vertical reduction compared to the control group. No significant differences were found between groups for palatal/lingual bone reduction or for changes in the overall bucco-lingual ridge width.

**Conclusions:**

The application of ACS combined with rhBMP-2 is an effective approach for mitigating buccal bone resorption after tooth extraction. This intervention is clinically significant as it preserves critical buccal plate height, potentially reducing the need for further augmentation prior to implant placement, even though it does not prevent overall ridge width collapse.

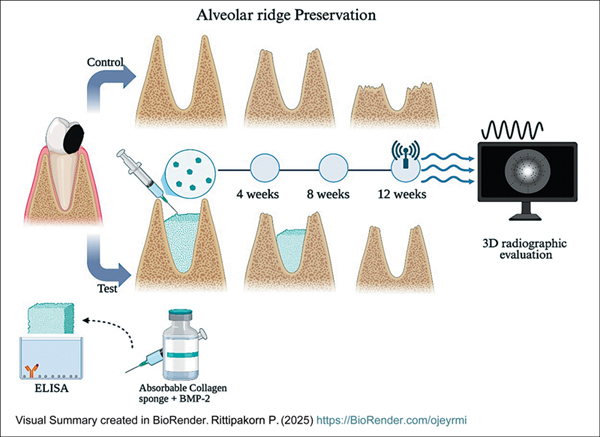

## Introduction

The healing process of the alveolar socket after tooth extraction initiates with the formation of a blood clot within the first 3 days, followed by substantial bone remodeling and mineralization within approximately 30 days. The dimensional alterations of the alveolar ridge subsequent to extraction have been documented extensively. Horizontal bone resorption of the alveolar ridge can range between 29 and 63%, and vertical resorption is typically reported between 11 and 22% within the first 6-month period following tooth extraction. 1 A study similarly reported that alveolar ridge width reduction can range from approximately 2.5 to 4.6 mm, with height loss varying between 0.8 and 3.6 mm when healing occurred without any intervention. 2 These dimensional changes predominantly occur within the first 3 months post-extraction, subsequently progressing at a slower rate. Alveolar ridge preservation (ARP) techniques, therefore, have become essential clinical approaches to minimize these dimensional changes, facilitate delayed implant placement, and maintain adequate ridge dimensions both horizontally and vertically. 2

Various methods are mentioned to preserve alveolar bone before an implant placement, including the use of allograft, xenograft, and alloplastic materials. 3 4 5 6 Autogenous dentin biomaterial processed from extracted teeth has recently shown promising osteoconductive and osteoinductive properties for ARP. 7 8 Biphasic calcium phosphate (BCP) has been developed as a carrier for bone morphogenetic protein-2 (BMP-2) 9 10 11 ; however, BCP has limitations in releasing sufficient BMP-2 during the early healing phase. 12 Absorbable collagen sponge (ACS) with BMP-2 is commercially introduced in orthopaedic spine surgery. 13 In oral and maxillofacial surgery, significantly greater bone augmentation has been observed in the ACS combined with 1.5 mg/mL recombinant human bone morphogenetic protein-2 (rhBMP-2) compared to the ACS alone in the extraction socket. 14 Additionally, the resorption of the collagen occurs through enzymatic hydrolysis. The collagenase concentration in the human saliva plays a role in the collagen degradation. 15 Furthermore, the most common clinical application of collagen usually is covering the primary wound for healing with optimum results.

The rhBMP-2 was clinically applied with collagen sponge for orthopaedic surgery. 16 However, the recommended dosage is extremely high (about 12 mg per ACS unit, which is approximately 37 mg of BMP-2 per gram of ACS sponge), which probably leads to adverse side effects. 17 Previous research indicated that a low-dose of BMP-2 can induce RUNX2, a crucial transcription factor for an early new bone formation. The optimum concentration of 10 µg/mL effectively induced osteoblast cell proliferation. 18 The potential of collagen sponge with rhBMP-2 in alveolar preservation was shown in Barboza et al and Jovanovic et al's research studies revealing a greater alveolar augmentation and bone formation with rhBMP-2/ACS. 19 20 Hunt et al further supported its use in a hyaluronan sponge carrier for bone reconstruction in alveolar ridge defects. 21 These studies collectively suggested that the use of collagen sponge with rhBMP-2 could be beneficial in alveolar preservation.

Despite evidence supporting the effectiveness of rhBMP-2 in bone regeneration, its application in dentistry remains underexplored due to the lack of specific guidelines, limited randomized controlled trials, varied clinical applications, and high costs. Furthermore, studies focusing on affordable and practical delivery systems for rhBMP-2 in ARP following tooth extraction are scarce. This study aims to evaluate the efficacy of a clinically applicable collagen sponge combined with rhBMP-2 for ARP. This pilot clinical trial seeks to determine whether this combination can effectively promote bone regeneration and maintain alveolar ridge dimensions after tooth extraction, offering insights into its potential as a practical and cost-effective solution for ARP in dental applications.

## Materials and Methods

### *In Vitro* Releasing Assay

**Fig. 1:**
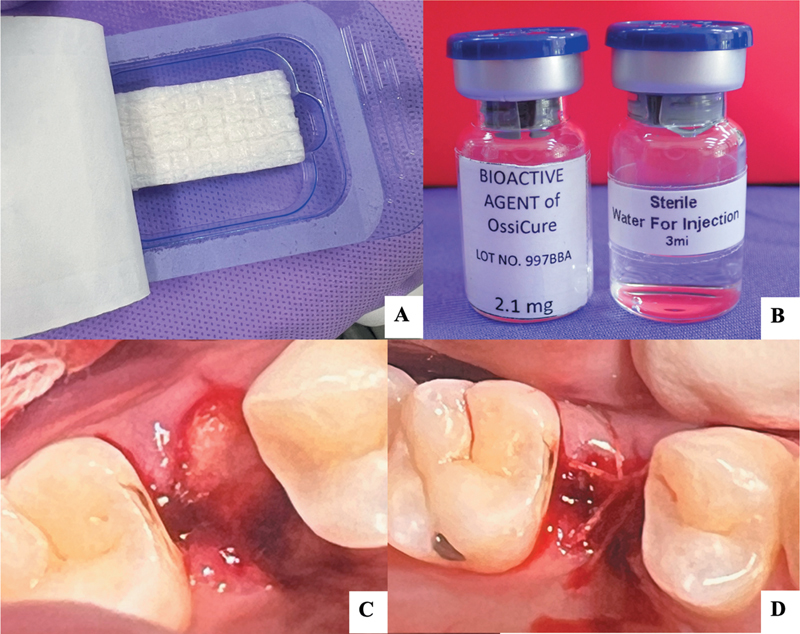
The absorbable collagen sponge (ACS) (Spon-Tiss, NSTDA, Thailand) and the rhBMP-2 protein (Ossicure, NSTDA, Thailand) were utilized in this study ( **A, B** ). The extraction socket immediately after the tooth was removed ( **C** ) and the rhBMP-2/ACS placed into the socket, followed by a resorbable suture ( **D** ). rhBMP-2, recombinant human bone morphogenetic protein-2.

ACSs (Spon-Tiss, NSTDA, Thailand) measuring 2.5 × 5 cm ^2^ were prepared as carriers for 2.1 mg of rhBMP-2 (Ossicure, NSTDA, Thailand; Fig. 1A, B ). Each ACS was individually cut to fit the extraction socket dimensions for subsequent release kinetics analysis. The rhBMP-2 solution was prepared at a concentration of 1.5 mg/mL in collagen sponge, with 0.2 mL loaded onto each carrier. Release kinetics were measured over 13 days by immersing the rhBMP-2-loaded collagen sponges in Dulbecco's Modified Eagle Medium (DMEM) supplemented with 2% heat-inactivated horse serum (HS) and incubating at room temperature. The use of 2% HS was to prevent the nonspecific adsorption of released rhBMP-2 onto the assay plate surfaces. The medium was replaced daily, and BMP-2 protein levels in the collected medium were quantified using enzyme-linked immunosorbent assay (ELISA; PeproTech, United States). A cumulative release curve was constructed based on the daily measurements.

### Study Design

**Fig. 2:**
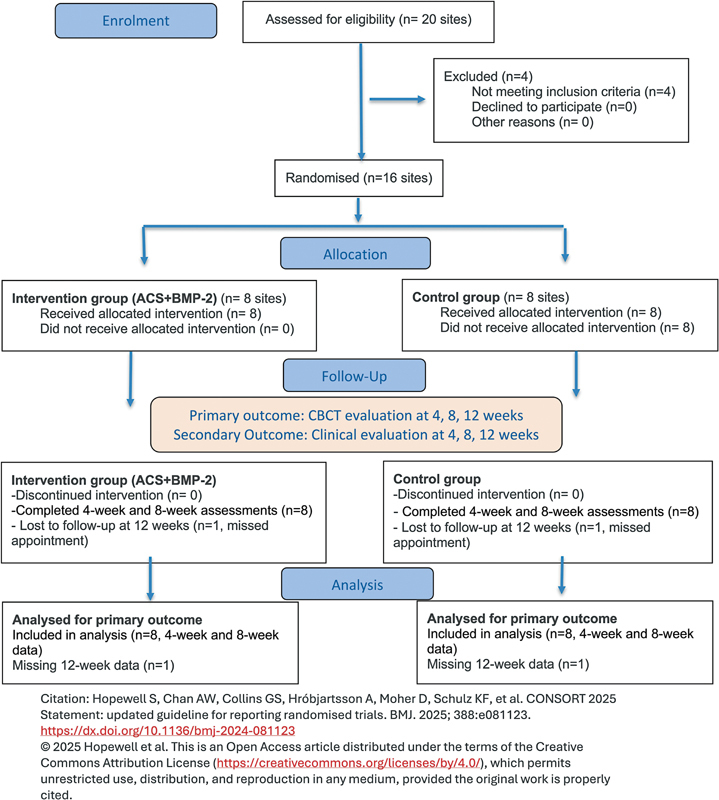
CONSORT 2025 flow diagram illustrating participant progress through the pilot clinical trial.

This pilot study was designed as a randomized controlled trial and was conducted in full accordance with the Declaration of Helsinki. Ethical approval was obtained from the Institutional Review Board of the Human Research Ethics Committee at Suranaree University of Technology (HREC-SUT), Thailand (Approval Number: EC-66-0105). All participants provided written informed consent prior to enrollment, and the trial was registered in the Thai Clinical Trials Registry (TCTR20240715009). The study followed CONSORT guidelines ( Fig. 2 ).

### Sample Size Calculation

Sample size calculation for this pilot study was based on feasibility considerations and informed by previous clinical trials with similar interventions, particularly the study by Coomes et al. 14 Coomes et al demonstrated that approximately 14 sites per group were sufficient to detect clinically significant differences with a statistical power of 80% at an alpha level of 0.05. Given the exploratory nature and practical constraints of this pilot study, these guidelines were used as a reference for determining the sample size.

### Participants and Randomization

A total 11 participants and 16 extraction socket sites (8 controls and 8 tests) were enrolled in this study. Randomization was performed at the site level, where each extraction site was individually allocated to either the treatment (ACS/rhBMP-2) or the control group. For participants requiring bilateral extractions, one side was randomly assigned to receive the intervention, while the contralateral side served as control. Participants with only a single extraction site had that site individually randomized to either the intervention or the control group. Due to the intra-patient control design, intra-class correlation coefficients for examiner calibration were not calculated.

### Treatment Protocol

#### Inclusion Criteria

- Patients who required extraction of single premolar tooth due to unrestorability (caries, fracture, or orthodontic treatment) and had more than 50% of buccal bone plate intact.
- Healthy adult patients (≥20 years) capable of providing informed consent.
- Extraction sites at least 2 mm of keratinized gingival tissue.
- Adjacent tooth to extraction site demonstrating mean bleeding on probing <20%. 7

#### Exclusion Criteria

- Active periodontal disease.
- History of smoking, radiotherapy, or chemotherapy within the past 5 years.
- Pregnancy or breastfeeding.
- Previous or current use of medications, e.g., steroids, antiresorptive drugs that affect bone remodeling phase, or known allergy to collagen.
- General contraindications to surgical treatment including current use of antiplatelet or anticoagulation drugs. 7

Throughout the entire study period, no orthodontic forces were applied to the teeth following premolar extraction, including in patients who had orthodontic brackets, to minimize confounding effects related to orthodontic treatment.

### Clinical Procedure

Local anesthesia (2% lidocaine with epinephrine 1:10 ^5^ in quantity; 1–2 cartridges) was administered. The buccal bone plate was carefully evaluated for any fenestrations or bone defects prior to extraction using transgingival bone sounding. The tooth was atraumatically extracted by a single surgeon using standard universal forceps. The extraction socket was then irrigated with 0.9% normal saline. In the experimental group, ACS soaked with 1.5 mg/mL rhBMP-2 solution was inserted 1 mm below the marginal bone, followed by a figure-of-eight stitch using 4–0 absorbable sutures (Vicryl 4–0; Ethicon, Norderstedt, Germany; Fig. 1C, D ). In the control group, the socket was left empty with no ridge preservation. Both groups followed the same postoperative care regimen receiving postoperative antibiotics including 500 mg amoxicillin and 400 mg ibuprofen every 8 hours for 5 days. The antiseptic mouthwash with 0.12% chlorhexidine gluconate was used for rinsing two times a day for 1 week. Sutures were removed after 2 weeks.

### Outcome Measurement

**Fig. 3:**
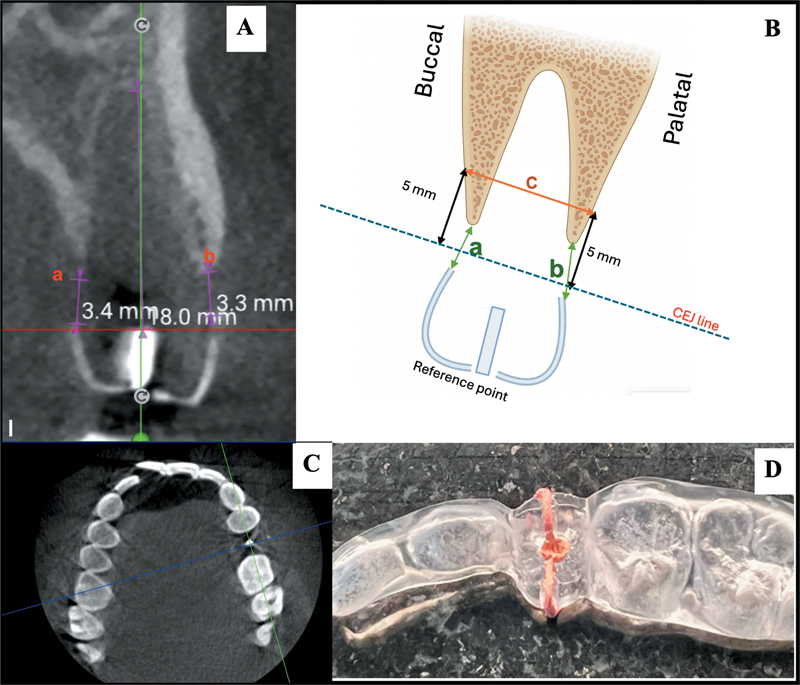
( **A** ) Measurement of vertical reduction at buccal height (a) and palatal height (b) using CBCT images. ( **B** ) Measurement protocol for bucco-palatal/lingual width (c). ( **C** ) The focal trough was set in the axial view to align with the dental arch and adjacent teeth. ( **D** ) Each measurement (a–c) was taken from a radiographic stent at specific reference points to ensure accurate and consistent evaluation of postextraction changes in alveolar ridge dimensions. CBCT, cone-beam computed tomography.

Clinical and radiographic examinations of the extraction sites were performed immediately after tooth extraction at baseline (T0), and subsequently at 4 weeks (T4), 8 weeks (T8), and 12 weeks (T12). Alveolar ridge dimensions were assessed using a radiographic acrylic stent in conjunction with cone-beam computed tomography (CBCT) images ( Fig. 3 ) acquired using the Acteon XMIND trium system (Acteon Group, Mérignac, France). CBCT measurement was achieved using viewing software.

The imaging protocol was set to standard mode, with the following exposure parameters: tube current 8 mA, tube voltage 85 kV, and voxel size 87.5 µm. A clear radiographic stent—identical in form to those used for implant planning—was fabricated for each study site. Three gutta-percha markers were embedded in the stent: one at the mid-occlusal point of the crown wax-up, and one each at the buccal and palatal mid-crown positions ( Fig. 3D ).

In each CBCT scan (T0, T4, T8, and T12), multi-planar reconstruction views were utilized. The three radiopaque markers defined the focal trough in the axial view, allowing alignment passing along the midline of the dental arch and in alignment with the adjacent teeth ( Fig. 3C ) and providing a reproducible reference line. Perpendicular distances from this line to the buccal and palatal crests were measured in the coronal view (a and b distances). The bucco-lingual ridge width was measured as the horizontal distance between these points (distance c) at a standardized level of 5 mm apical to the cementoenamel junction of the adjacent tooth, as visualized in the sagittal CBCT view ( Fig. 3A, B ).

All radiographic measurements were performed twice by a single calibrated examiner who was blinded to the group allocation, ensuring both reliability and intra-examiner consistency.

### Statistical Analysis

The data were presented as mean ± standard deviation. Demographic data were analyzed using descriptive statistics. The Shapiro–Wilk test was conducted to assess the normality of the data. A linear mixed model was applied to compare follow-up periods between groups. All statistical analyses were performed using RStudio (Version 4.4.1; Posit, PBC, United States). A significance level of *p* <0.05 was considered statistically significant.

## Results

### rhBMP-2 Cumulative Release

**Fig. 4:**
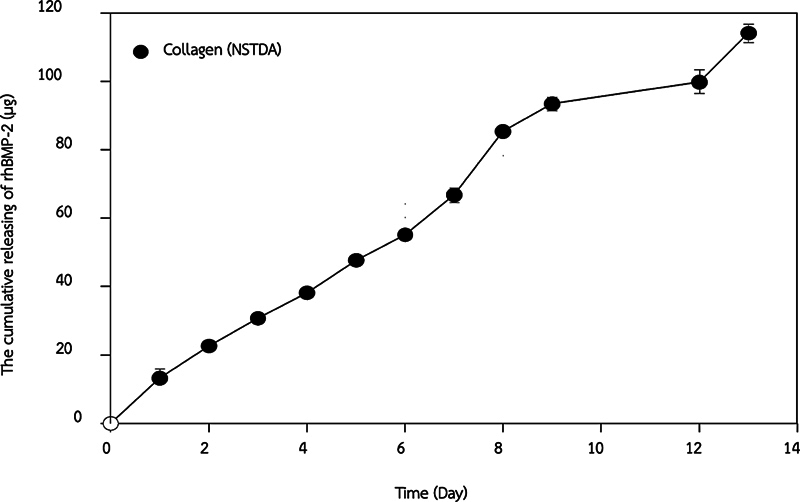
The releasing kinetics of rhBMP-2 from collagen sponge over a 13-day period. rhBMP-2, recombinant human bone morphogenetic protein-2.

The releasing kinetics of rhBMP-2 from collagen sponge over 13 days was analyzed using ELISA (Peprotech, United States). The cumulative release of rhBMP-2 from the collagen sponge was gradual, with 117 ± 5.18 µg released over 13 days, representing 7.28% of the initial loaded amount ( Fig. 4 ).

### Clinical Evaluation

All patients who received the ACS/BMP-2 group have no postoperative complications, such as impaired socket healing, swelling, or dry socket. The collagen sponge was completely resorbed in the first month. The soft tissue healing in both groups was complete at the first follow-up period.

### Radiographic CBCT Assessment

Eleven participants (3 males, 8 females; mean age 28.5 ± 13.9 years) contributed 16 extraction sites that were randomized to the control (empty socket) or the rhBMP-2 group. One site per group was lost to follow-up at T12. In the control group, the mean vertical reduction of the buccal bone plate was 1.53 ± 0.54 mm at T4, progressing to 2.62 ± 1.14 mm at T8, and 3.30 ± 1.19 mm at T12. In contrast, the ACS/rhBMP-2 group showed significantly less bone loss. At the 12-week follow-up, the ACS/rhBMP-2 group demonstrated a statistically significant preservation of buccal bone height, with 1.80 mm less reduction compared to the control group (95% confidence interval: 0.64–2.95 mm; *p* = 0.0049). Palatal/lingual bone resorption was generally comparable between the groups; however, the control group showed significant palatal/lingual resorption between the T4 and T12 evaluations.

Regarding the bucco-lingual width dimension, significant reduction was observed in the control group, starting at 4 weeks and continuing through 12 weeks (T4: −0.68 ± 0.48 mm, T8: −1.10 ± 0.78 mm, T12: −1.52 ± 0.87 mm). The ACS/rhBMP-2 group exhibited a relatively stable ridge dimension over the same periods (T4: −0.48 ± 0.56 mm, T8: −1.14 ± 1.40 mm, T12: −1.36 ± 1.32 mm). Time significantly influenced the bucco-lingual width reduction ( *p* < 0.05), but no significant interaction effect between group and time was observed.

**Fig. 5:**
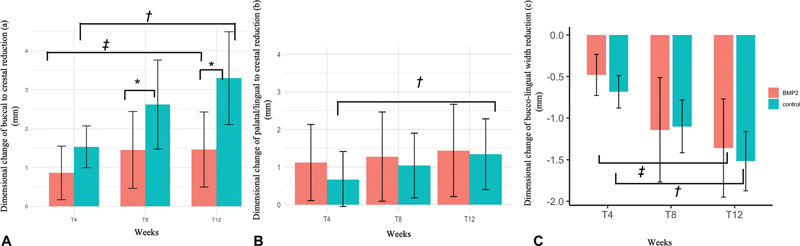
Dimensional changes of the alveolar ridge in the control and experimental groups. Bar graphs ( **A** ) and ( **B** ) illustrate the mean reductions in crestal height over time (4, 8, and 12 weeks) for buccal and palatal aspects at the extraction sites, respectively. Bar graph **(C** ) represents horizontal bone reduction, showing the mean changes in bucco-lingual width over time. Statistically significant differences ( *p* < 0.05) are indicated by *, **, †, and ‡.

Overall trends of alveolar ridge dimensional changes in both groups are detailed in Tables 1 and 2 and illustrated in Fig. 5 .

**Table 1:** Mean alveolar ridge dimensional changes in the control and experimental group (mm ± SD) ( *p* < 0.05)

Dimensional change	Group	4 weeks (T4)	8 weeks (T8)	12 weeks (T12)	*p* -Value
**a (buccal reduction)**	Control	1.53 ± 0.54 a	2.62 ± 1.14 b	3.30 ± 1.19 b	<0.01
	ACS/BMP-2	0.86 ± 0.69 c	1.45 ± 0.98	1.46 ± 0.96 c	>0.01
**b (lingual/palatal reduction)**	Control	0.67 ± 0.75 a	1.04 ± 0.86	1.34 ± 0.94 a	<0.05
	ACS/BMP-2	1.12 ± 1.01	1.27 ± 1.19	1.44 ± 1.23	>0.05
**c (bucco-lingual reduction)**	Control	−0.68 ± 0.48 a	−1.1 ± 0.775	−1.52 ± 0.87 a	<0.05
	ACS/BMP-2	−0.48 ± 0.56 c	−1.14 ± 1.40	−1.36 ± 1.32 c	<0.05

Abbreviations: ACS, absorbable collagen sponge; BMP-2, bone morphogenetic protein-2; SD, standard deviation.

Note: Data are presented as mean ± SD. See Table 2 for detailed statistical comparisons of buccal reduction between groups.

a Significant intragroup difference between T4 and T12 in the control group ( *p* < 0.05).

b Statistically significant difference between groups at the same time point ( *p* < 0.05).

c Significant intragroup difference between T4 and T12 in the ACS/BMP-2 group ( *p* < 0.05).

**Table 2:** Between-group comparison of buccal reduction over time

Time point	Mean difference (mm) (control vs. ACS/BMP-2)	95% confidence interval (CI) for the difference	Effect size (Cohen's *d* )	*p* -Value
**4 weeks (T4)**	0.79	[−0.35, 1.93]	1.82	0.1601
**8 weeks (T8)**	1.27	[0.09, 2.45]	2.94	0.0360
**12 weeks (T12)**	1.80	[0.64, 2.95]	4.15	0.0049

Abbreviations: ACS, absorbable collagen sponge; BMP-2, bone morphogenetic protein-2.

Note: Mean difference > 0 indicates less bone resorption in the ACS/BMP-2 group.

## Discussion

The buccal reduction in the ACS/rhBMP-2 group was significantly less than the control group at T8 and T12. However, palatal/lingual reductions and bucco-lingual width were similar between the two groups. Specifically, the control group showed a buccal bone reduction of approximately 3.3 mm after 3 months. It is well-documented that dimensional alterations of the alveolar ridge occur predominantly during the initial 3-month post-extraction period, resulting in substantial horizontal resorption of approximately 50% of the original ridge width and an average vertical loss of around 1.24 to 1.79 mm. These initial changes are particularly pronounced on the buccal aspect, emphasizing the importance of timely interventions to mitigate bone resorption and establish optimal conditions for subsequent implant placement. 2 22 23 The observation that the ACS/rhBMP-2 group preserved an average of 1.80 mm more buccal bone than the control group at T12 is not only statistically significant ( *p* = 0.0049) but also clinically meaningful. This therapeutic effect was notably large (Cohen's *d* = 4.15). From a clinical standpoint, this degree of bone preservation is critical, as maintaining buccal plate thickness can reduce or eliminate the need for subsequent guided bone regeneration procedures during implant placement. This result aligns closely with findings by Fiorellini et al, who reported that 1.5 mg/mL of rhBMP-2 with an ACS significantly promoted bone formation in extraction sockets. 24 Furthermore, a recent systematic review highlighted that the application of rhBMP-2/ACS in extraction sockets notably reduced horizontal alveolar ridge resorption, particularly when used at a higher dosage of 1.5 mg/mL, suggesting a dosage-dependent efficacy in preserving alveolar dimensions. 25 Similarly, a recent systematic review highlighted that rhBMP-2/ACS is effective in reducing horizontal alveolar resorption, suggesting a dosage-dependent efficacy. Our findings collectively support the role of ACS/rhBMP-2 as an effective approach for minimizing post-extraction alveolar ridge resorption. 26

The rhBMP-2 with collagen sponges promotes osteogenic differentiation of mesenchymal cells and supports bone regeneration. Clinical studies comparing xenograft/ACS/rhBMP-2 to bovine bone alone found no significant ridge height difference but did show greater ridge width preservation and buccal bone height gain (1–2 mm) in the rhBMP-2 group. 27 The rhBMP-2 exhibits dose-dependent effects influenced by its carrier system. The protective effect of low-dose rhBMP-2 was restricted to the buccal plate for two hypothesized reasons. Anatomically, the buccal wall is a <1-mm-thick bundle bone that remodels rapidly after extraction, whereas the thicker palatal/lingual plate shows minimal baseline loss; thus, any extra osteogenic stimulus yields little additional gain on the palatal side. 1 28 Because horizontal ridge width depends on the preservation of both buccal and palatal cortex, selective buccal preservation alone is insufficient to prevent the overall bucco-lingual collapse observed in both groups at T12. Materially, rhBMP-2 delivered by the collagen sponge diffuses only a short distance and promotes intramembranous bone formation where a blood clot can be stabilized. The palatal side is already space-filled by native bone, leaving less room for de-novo formation than the buccal defect. These factors together explain why significant height reduction was averted buccally but not palatally, and why ridge width remained comparable between groups.

The collagen sponge combined with xenografts has been widely used for ARP. This combination prevents alveolar ridge collapse horizontally and blocks soft tissue infiltration into the lower socket. 4 While xenogenic bone grafts combined with BMP-2 are commonly used for bone augmentation, ARP applications primarily employ alloplastic bone grafts, such as hydroxyapatite or β-tricalcium phosphate, due to their carrier properties. 11 29 However, a major limitation of xenograft materials is their notably slow resorption rate, which can extend for periods as long as 9 months or even longer. This prolonged degradation period might result in persistent residual particles within the grafted site, potentially interfering with complete bone regeneration and integration. 30 31 CBCT studies have shown that collagen sponges alone, applied to fresh extraction sites using an open-wound technique, effectively reduce socket collapse. 32 Collagen sponges serve as an effective carrier, enabling sustained release at low doses. 26 33 A comparison with autogenous bone grafts also revealed similar bone gain (∼3.5 mm) over 6 months. Furthermore, a comparison between autogenous bone grafts and 1.5 mg/mL rhBMP-2/ACS for horizontal ridge augmentation showed no significant differences between groups over 6 months, with both achieving approximately 3.5 mm of bone gain. 34

The releasing kinetics of rhBMP-2 from the ACS was measured for *in vitro* . This study reports that approximately 100 µg of rhBMP-2 is released over 13 days at a gradually decreasing rate. This release activates osteogenesis during the early phase of bone remodeling. 35 The optimal rhBMP-2 dose for bone formation ranges from 0.8 to 500 µg/g of the delivery system, with 100 µg providing balanced bone formation on titanium-coated implants. 36 Similarly, 100 ng/mL of rhBMP-2 on ACS can sustain release over 10 days, significantly inducing osteoblastic differentiation *in vitro* . 37 While 2% HS was used in the *in vitro* study, the albumin-driven desorption mechanism of rhBMP-2 is species-independent; nevertheless, absolute release in human plasma may be marginally faster owing to higher protease activity. Future work will include parallel assays in human serum to confirm this observation. Several factors influence the incorporation of rhBMP-2 into ACS. The hydrophilic double-extended homodimer form of rhBMP-2 has the least binding affinity to ACS, suggesting that extending the soaking time before implantation could enhance protein incorporation and improve bone regeneration. 38 However, collagen sponges have inherent limitations, such as rapid degradation and poor mechanical strength due to their high porosity. Strategies like cross-linking have been explored to improve these properties, enhancing structural integrity and prolonging scaffold degradation time. 39

A limitation of our *in vitro* release study is that it was conducted at room temperature. While this provides a fundamental understanding of the release profile, the absolute release rate *in vivo* may be faster due to temperature-dependent effects on molecular diffusion and the biodegradation of the collagen carrier. 40 The primary limitation is the relatively small sample size, typical for pilot studies, which may limit the strength and generalizability of our findings. Additionally, patient-specific variables such as individual healing capacities and surgical technique variability might influence outcomes. Finally, the short-term follow-up may not fully represent long-term clinical outcomes or complications. Future studies should include larger sample sizes and longer follow-up periods to validate these preliminary results. Although most extraction sites were consistently located in the anterior maxilla, one patient had bilateral mandibular premolars extracted, serving as both intervention and control. While this design reduced inter-individual variability, it might introduce variability due to differences in bone remodeling between jaws. Additionally, exploring combination therapies and novel biomaterials may provide further insights into effective ridge preservation strategies.

## Conclusions

The application of an ACS combined with rhBMP-2 (ACS/rhBMP-2) is an effective strategy for ARP, specifically for maintaining buccal bone height following tooth extraction. Our findings demonstrate that this combination therapy resulted in a statistically and clinically significant preservation of 1.80 mm of buccal bone at 12 weeks compared to the control sites. The sustained, low-dose release of rhBMP-2 from a clinically approved collagen sponge promoted early osteogenesis without adverse events, highlighting its practicality and safety in routine dental practice. These results support the adoption of ACS/rhBMP-2 for cost-effective ARP and lay the groundwork for larger clinical studies to refine dosing strategies, expand indications, and integrate this approach into standard post-extraction.
